# SQUARE-IT: a proposed approach to square the identified research problem in the literature with the objectives, the appropriate clinical research question, and the research hypothesis

**DOI:** 10.1186/s12874-025-02468-7

**Published:** 2025-01-27

**Authors:** Martin Alfuth, Jonas Klemp, Annette Schmidt, Lukas Streese, Nikolai Ramadanov, Robert Prill

**Affiliations:** 1 Faculty of Health Care, Therapeutic Sciences, Niederrhein University of Applied Sciences, Reinarzstr. 49, Krefeld, 47805 Germany; 2 Department of Nephrology, Medical Faculty, University Hospital Düsseldorf, Heinrich-Heine-University Düsseldorf, Düsseldorf, Germany; 3 Center of Orthopaedics and Traumatology, University Hospital Brandenburg/Havel, Brandenburg Medical School Theodor Fontane, Brandenburg, Germany; 4 Faculty of Health Sciences Brandenburg, Department of Orthopaedics and Traumatology, Brandenburg Medical School Theodor Fontane, Brandenburg, Germany; 5 Center of Physiotherapy, Center of Orthopaedics and Traumatology, Center for Joint Replacement West-Brandenburg, University Hospital Brandenburg/Havel, Hochstrasse 29, Brandenburg / Havel, 14770 Germany

**Keywords:** Evidence, Guidance, Healthcare, Quality, Research

## Abstract

The purpose of this article is to design and introduce the SQUARE-IT approach to help scientists and clinicians in research to align important research problems with the objectives, the appropriate clinical research questions to be answered, and the research hypotheses to be investigated in medical and therapeutic specialties. Research ideas can be generated primarily through simple methods such as brainstorming and mind mapping. However, transforming ideas into a valid research question is not as easy as it may seem, as the mere presence of an idea does not guarantee that the researcher has already uncovered existing knowledge on a particular topic or identified the actual research problem. Therefore, the SQUARE-IT items are developed, described, and critically discussed with reference to the scientific literature. They ask whether the identified research problem is ‘Specific’, ‘Quantifiable’, ‘Usable’, ‘Accurate’, ‘Restricted’, ‘Eligible’, ‘Investigable’, and ‘Timely’. Before formulating the focused clinical question, SQUARE-IT can be used as a preparatory step to enable researchers to organize the relevant information that has been explored to date and to assess whether additional information is needed, thereby identifying current research gaps. In addition, it should facilitate the effectiveness and efficiency of evidence-based practice to ensure high quality patient care. Using SQUARE-IT as a framework, further elaboration of the approach and addition of other aspects are warranted to advance the discussion and improve methods of evidence-based practice in medical and therapeutic specialties for quality improvement of patient care.

## Background

**Fig. 1:**
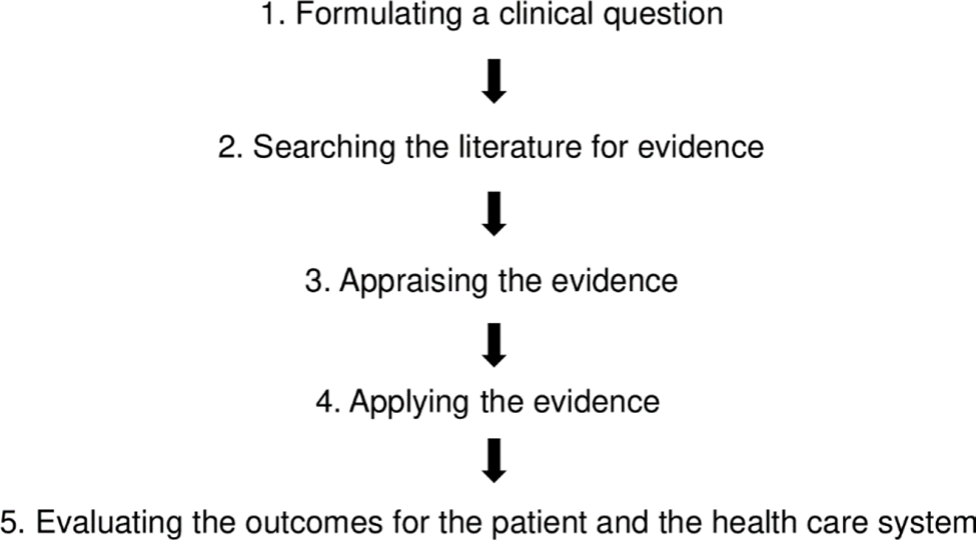
The five steps of Evidence-Based Practice (EBP)

Evidence-based practice (EBP) refers to the integration of the most up-to-date and reliable evidence from systematic research with effective clinical reasoning and individual clinical expertise [1–3]. This approach takes into account patient characteristics, individualization based on the patient`s situation, and patient preferences [1, 3]. Rather than replacing individual clinical expertise, the incorporation of EBP into clinical practice aims to support clinical decision-making processes [4]. The utilization of EBP is paramount in ensuring high-quality patient care [5], despite the potential challenges of being time-consuming and demanding [1]. The process of EBP consists of five steps, which are shown in Fig. 1 and described in detail elsewhere [1, 3, 6]. 

A well-formulated interventional related clinical question is essential in conducting evidence-based clinical practice [7], and can be formulated using the PICO-format, which consists of four elements: P (Problem/Patient), I (Intervention), C (Comparison/Control), and O (Outcome(s)) [8]. The purpose of the PICO-format is to provide an answer that supports a clinical decision, or for a researcher to design and configure the research to be completed [9]. Additionally, the letter T can be included in the format to indicate the appropriate follow-up time for assessing the outcomes [10, 11]. The research question should fulfill defined attributes, which have been summarized to the FINER approach [12]. Thereby, F stands for ‘Feasible’, I for ‘Interesting’, N for ‘Novel, E for ‘Ethically’ sound, and R for ‘Relevant’ [13]. To obtain the best available evidence, a combination of clinical experience, input from patients/clients and caregivers, local context, setting and research should be considered [2]. For the latter, there are various checklists, statements, recommendations, and reporting guidelines, e.g. for original studies and reviews. These resources aim to justify new clinical studies, address research gaps, and prevent to produce insufficient research, which is likely to contribute to research waste [14, 15]. 

However, these tools do not address an important step in the process. When a clinician, physical therapist, student, or aspiring researcher sets out to formulate novel ideas for original research, there is currently no streamlined and systematic approach available [16]. Most research ideas arise from an individual’s perception of knowledge gaps related to a scientific problem or clinical dilemma. Particularly in the clinical setting, students and aspiring researchers often look to their supervisors to fill knowledge gaps in order to engage in clinical decision making. In clinical research, for example, graduate students are increasingly required to take responsibility for identifying research gaps in order to formulate a research question. These gaps may result from a lack of clear conclusions or inadequate results from previous studies [13], or from the selection of an inappropriate study type that cannot be corrected after the study begins and results in flawed methodology [17]. In addition, the appropriate methodology of a study depends on the objectives, study population, study design, techniques, sampling, and statistical procedures used [17], and can be a source of research gaps if not conducted correctly.

**Fig. 2:**
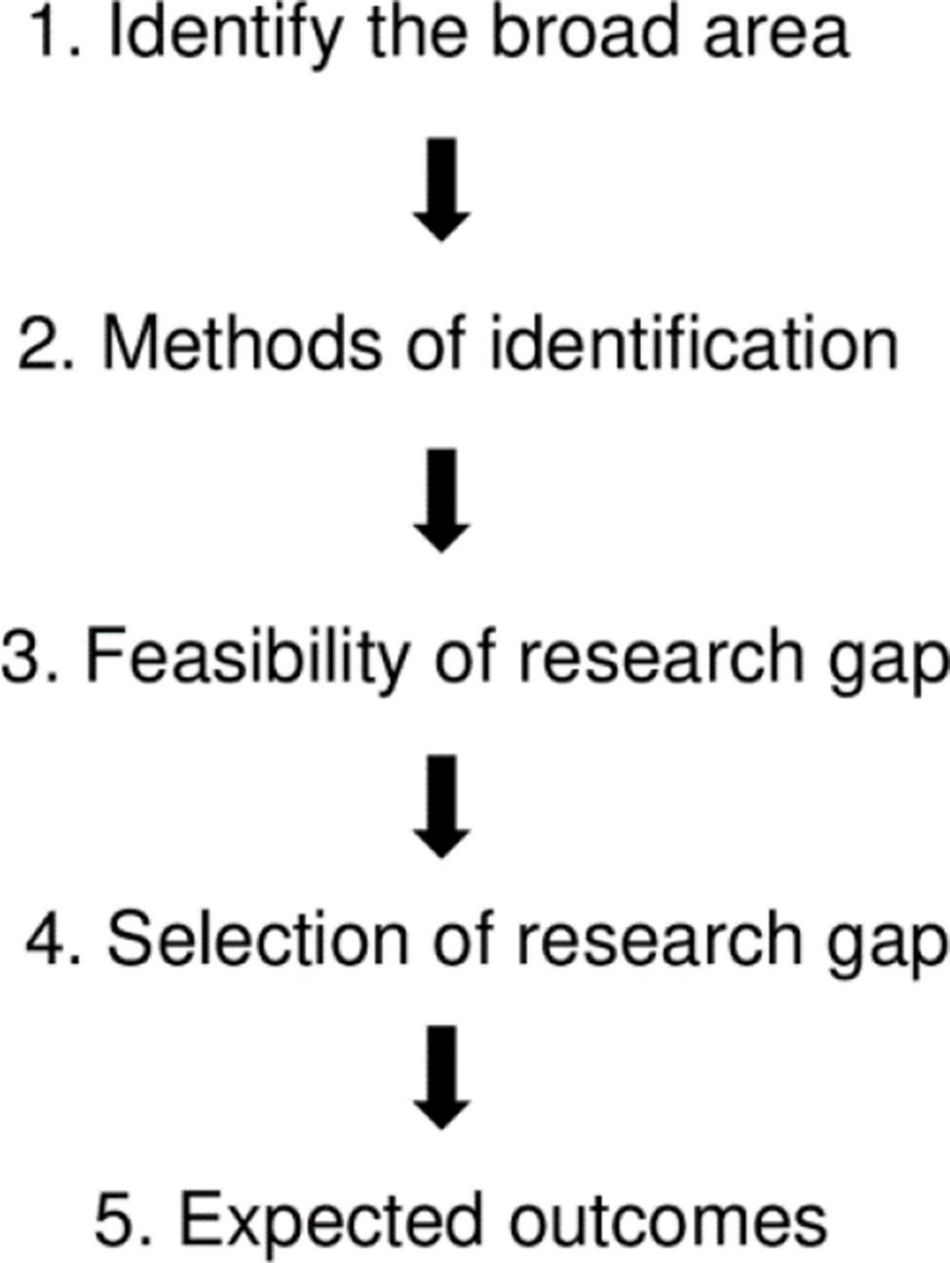
Process of research gap analysis according to Farooq [23].

Identifying and introducing the research problem in clinical settings or literature, associated with the significance of the research topic, is crucial before systematically reviewing the literature of a specific research question and carrying on with the research process in terms of formulating purposes/objectives and hypotheses [18]. However, the process of systematically discovering the research problem and aligning it with objectives, research questions, and hypotheses remains a challenge [7, 19]. This challenge was previously acknowledged by McGuire [20] in the field of psychology. He highlighted the lack of predefined procedures or guidelines to generate research questions and hypotheses. Some authors noted that there is no systematic process for identifying research problems and provided frameworks for identifying research gaps from systematic reviews [21, 22]. Farooq [23] introduced a helpful process of research gap analysis for the social sciences, which includes five steps (Fig. 2).

In addition, the author developed a conceptual model of the research gap, which presents sources and methods for identifying and analyzing a research gap [23]. These are presented in Box 1.

**Table:** 

Box 1. Sources and methods for identifying and analyzing a research gap^23^	
• Citation analysis • Content analysis reports • Meta-analysis • Systematic reviews • Future research and limitations	

In particular, the last three sources are very important for identifying clinical research problems, because advice and recommendations from authors of original research articles, systematic reviews, and meta-analyses are the main triggers for initiating needed research. When the research gap is identified, it leads to problem identification, which Farooq [23] defines as the research question, which we agree is not the end of the research gap analysis. The frameworks, processes and models focus primarily on identifying and characterizing gaps without aligning them with the study objectives, the focused research question, and the hypothesis, which is strongly required for clinical research.

Therefore, the purpose of this article is to design and introduce the SQUARE-IT approach to the scientific community, especially to aspiring and novice clinicians and researchers [24]. SQUARE-IT is intended to be a useful tool for identifying significant research problems and for aligning these newly identified research problems with appropriate objectives, clinical research questions, and hypotheses in primary and secondary clinical research, i.e., original studies and systematic reviews with or without meta-analyses.

## Defining the term ‘research problem’

A ‘research problem’ [25–27], also called ‘research gap’ [2, 22], arises when absence or insufficiency of data limit the researchers ability to derive a meaningful conclusion about a specific topic. It is important to focus on precisely formulated research questions to avoid confusion and waste of resources. Each piece of research usually starts with the definition of the research problem [28]. The research problem itself typically consists of the purpose, which asks why the research question is important, and one or more objectives, which ask whether the researcher will know whether they have satisfactorily addressed the research problem [29, 30]. Subsequently, the focused research question(s) and hypothesis or hypotheses can then be formulated based on the objective(s) [28]. In the literature, there is often no clear distinction between the research problem/gap and the research question, which we believe should be the case, because if you go straight from the general research topic to the research question, it is not clear that the questions are relevant to the problems within the field/setting.^16^ A problem in everyday life is something that one tries to avoid, but a research problem is something that the researcher seeks out [31], in order to address it by asking the appropriate questions. Researchers are more likely to develop relevant research questions if they focus on the research problems first [16]. The research question is an uncertainty about a problem that arises in daily research activities and clinical practice and can be investigated, evaluated, and analyzed to provide important information [7]. Therefore, the existing research problem should be identified and formulated in more than one sentence, moving from complexity to specificity. Subsequently, the research question should be focused and yet simple [32] and clearly stated [13] in a single sentence, making clear what the study will investigate, in which population, using appropriate outcomes that should be based on the identified research problem. It should be in the form of a question and should be complete in itself rather than being broad [13, 32]. 

### Rationale for the implementation of the SQUARE-IT approach

During the initial stages of the research process, there are various approaches available to identifying a research topic. While ideas may be abundant, they still need to be refined into valid research questions [13]. Sources of ideas may be the practitioner’s or researcher’s professional environment, their own experiences and interests, observations, professional discussions, previous study results, indicating a lack of conclusions or insufficient outcomes [13]. However, young scientists or clinicians embarking on their scientific careers may not readily have access to all these sources. Research ideas can be primarily initiated by simple methods, such as brainstorming [13] and mind mapping [28]. Nevertheless, transforming ideas into a valid research question is not as straightforward as it may seem [19], as the mere presence of an idea does not guarantee that the researcher has already uncovered existing knowledge about a particular topic [16], or identified the actual research problem. A consistent definition and organization of the research problem are important to provide input for evaluating the effectiveness of different methods and procedures, which is urgently needed to generate new evidence to improve clinical practice [24]. At this early stage of research, commonly referred to as the planning stage, researchers engage in an extensive research of the available literature to obtain important information before beginning the work on their actual research project [17], including the formulation of the objectives, research question(s), and hypotheses. Hence, it can be beneficial to implement SQUARE-IT as a preparatory step, enabling researchers to organize the pertinent information that has been explored thus far and assess whether additional information is required, thereby identifying current research gaps. Researchers are advised to focus on at least four main types of information from current literature and practice: theory, facts, opinions, and methods [16]. 

Once researchers have identified a research problem, they must square it with the objectives, research question, and hypothesis of the planned study. The letters of the acronym SQUARE-IT represent the items ‘Specific’, ‘Quantifiable’, ‘Usable’, ‘Accurate’, ‘Restricted’, ‘Eligible’, ‘Investigable’, and ‘Timely’. Table 1 presents the short definitions of the items in the context of the discovery and evaluation of the research problem from the initial ideas. The following section details the meaning of each item and how the items can help to appropriately square the research problem with the objectives, research question, and hypothesis.

**Table 1:** SQUARE-IT: short definition of each term

Item	Definition
**S**pecific?	Is your research idea specific and does it pose a relevant problem at all?
**Q**uantifiable?	Is the research problem quantifiable based on clear independent and dependent variables?
**U**sable?	Are the outcome variables usable for clinicians and researchers, and patients?
**A**ccurate?	Is the identified research problem described accurate enough to clearly define the study objectives?
**R**estricted?	Can the discovered research problem and the variables be restricted reasonably?
**E**ligible?	Is the research problem eligible?
**I**nvestigable?	Can the research problem lead to facts in health care?
**T**imely?	Is the research problem timely and realistic in terms of the planned start, timeframe, and completion of data collection and publication of results?

## Detailed definition of each SQUARE-IT item

The item ‘Specific’ of the SQUARE-IT approach asks whether there is a specific, obvious, explicit, relevant and unambiguous research problem/gap in literature or clinical practice regarding the research topic. The identification of a clear and unambiguous problem to be investigated is a crucial step in establishing a research agenda [22]. The identified problem, which may be subject to challenge or improvement can arise from various source such as theory (that needs to be reevaluated regarding its validity or that may be novel), from existing facts (that may be represented by the current evidence), or from opinions (that may be advocated by experts of a certain clinical arena or methods) [16]. Researchers who seek to identify a specific research problem can explore research gaps within the conclusions of recent systematic reviews [22, 25, 33–35], qualitative and quantitative studies [24], or within their area of interest. Many studies explicitly highlight research gaps and recommend further research projects, including the appropriate study design, in their conclusions and outlook sections. Furthermore, researchers should be driven to uncover research gaps based on their clinical experience, necessitating an extensive literature search to confirm the potential existence of research gaps.

A way of moving from research ideas to a specific problem, and then to square the problem with the clinical question and hypothesis, is provided by Carter & Lubinsky [16] and includes the following sets, with examples or explanations, which are shown in Box 2.

**Table:** 

Box 2. Sets, with examples or explanations, to square the research problem with the clinical question and hypothesis^16^	
1. Topic identification and selection (e.g., “I want to do something with the knee”) 2. Problem identification and selection (e.g., studying a well-known clinical phenomenon in the context of a new population) 3. Theoretical framework identification and selection (after a problem is selected, it needs to be placed into a theoretical framework that will allow it to be viewed in relation to other research) 4. Question identification and selection (e.g., based on the problem in set 2.: “How does clinical depression present itself in individuals with acquired spinal cord injury?”)	

In this process of specifying research ideas, researchers should first be creative enough to generate many ideas, and then selective enough to focus on a limited number of ideas for further study [16]. 

The item ‘Quantifiable’ assesses whether a research problem is well-defined, quantifiable, and can be verified through the selection of appropriate independent and dependent variables. Research studies should include all important and relevant independent variables. An accurate assessment of the variables should be performed to generate robust results [36]. Including just one independent variable may result in only a partial description of the dependent variable, which represents a research problem/gap to be identified and addressed by the researcher. Different independent variables should be combined with a dependent variable to avoid missing important explanations of the dependent variable. The outcome (dependent) variables are considered as measurable endpoints of the objectives that will be observed throughout the study [13]. The occurrence (or non-occurrence) of the outcomes reveals that the predefined objective has been achieved. The outcome variables should be derived from - and consistent with - the study objectives. Examples of defining independent and dependent variables as part of the development of identifying a new research problem are provided by Carter & Lubinsky [16] and shown in Box 3.

**Table:** 

Box 3. Examples of defining independent and dependent variables as part of the development of identifying a new research problem^16^	
• Extending previous work by modifying aspects of the independent variable (e.g., “Would the same result have been achieved if speech therapy sessions were conducted more frequently?”) • Extending previous work by adding new dependent variables (e.g., “Does aquatic therapy for individuals with knee osteoarthritis improve participation levels and health-related quality of life, in addition to its established impact on impairment measures such as strength and range of motion?”)	

The ’Usable’ item examines whether the outcome variables that need to be quantified in the context of the research problem are usable by clinicians, researchers, and patients. Research findings should contribute to the existing body of evidence, enhancing the understanding of specific outcomes and contributing to the overall knowledge in the field [37, 38]. Scientific evidence in health care needs to be translated into practice to ensure rapid implementation and to reduce the gap between research and practice. To facilitate this, reporting guidelines for implementation strategies are provided, such as the ‘Standards for Reporting Implementation Studies (StaRI)’ guidelines or the Action, Actor, Context, Target, and Time (AACTT) framework introduced elsewhere [39]. Successful implementation of one’s own research findings can be achieved by aligning implementation strategies with implementation outcomes, which should be taken into account when specifying the research problem, objectives, and research question. Additionally, investigators should evaluate the clinical relevance of the results to patients. Clinical relevance refers to a significant improvement in measurement or treatment and should not be conflated with statistical significance [40]. 

The item ’Accurate’ asks whether the identified research problem is precisely described to clearly define the study objectives. Research problems are often multifaceted [41]. Therefore, researchers should articulate their ideas and existing evidence accurately and clearly [42], especially when trying to communicate complex ideas [27]. These ideas and current evidence should be incorporated into well-defined study objectives and clinical questions, as the reliability and validity of study results depend on the overall study design. This also includes reproducible methodology, careful data collection and analysis, and clear reporting of results [43, 44]. Within this item, the researcher should consider the PICO(T) format in advance so that the research problem can be more easily aligned with the clinical question and the hypothesis, which is considered to be the researcher’s reasoned estimate of the outcome of the research.

The item ’Restricted’ investigates whether the identified research problem and the variables can be reasonably confined, enabling control and differentiation from other research problems. Restricting, narrowing or limiting the clinical research problem can be achieved by using some strategies, including ‘aspect’, ‘components’, ‘methodology’, ‘place’, ‘relationship’, ‘time’, ‘combination’ [31]. ‘Aspect’ involves focusing on a specific facet of the research problem. ‘Components’ entails breaking down the primary variable into smaller parts to facilitate more accurate analysis. This allows the researcher to gain a more thorough understanding of the problem before proceeding to other steps in the research process [41]. ‘Methodology’ is the process of gathering information so that the amount of interpretive analysis required to address the research problem can be minimized [31]. This is where Kuhn’s [45] three methods of ‘isolate and structure’, ‘magnify the problem’, and ‘search for theory’ can be applied. The methods are described in Box 4.

**Table:** 

Box 4. Application of Kuhn’s methods^45^	
1. Isolate the problem from other external factors to gain a better understanding of the problem itself 2. Focus on a particular isolated part or parts of the problem to gain a better understanding of that particular isolated part of the problem 3. Conduct a complete literature review with the primary goal of finding enough relevant theory and research to formulate a well-structured argument from which the particular research questions can be derived	

‘Place’ means reducing the geographical unit of analysis in order to narrow the focus. ‘Relationship’ refers to how certain variables are related to each other. ‘Time’ refers to the appropriate timing of the study period and the timely start of the study and reporting of results after completion of data collection. ‘Type’ means focusing the research problem on a particular class of people, places, or phenomena. ‘Combination’ recommends using two or more of the aforementioned strategies to narrow the research problem [31], and to help focus it precisely on the research question.

The item ‘Eligible’ assesses whether the research problem and variables are eligible in terms of your research interests, research vision, beliefs, desires, and needs (personal factor) or the existing evidence in the field and priorities for future research, e.g. according to the James Lind Alliance (https://www.jla.nihr.ac.uk/), agreed by patients, funders, supporters, collaborators, caregivers, or policy makers (stakeholder factor); feasibility of the research; ethical standards, including Good Clinical Practice, which is the international standard for the design and conduct of clinical trials to ensure ethical and scientific integrity [46], and the EU General Data Protection Regulation [47]. Good research begins with intrinsic motivation for a specific area of research, protecting against discouragement and disinterest in the research as it progresses [41]. Then the researcher should begin to ask questions about the topic that will lead to the information needed to answer them. Suggested questions are ‘who’, ‘what’, ‘when’, ‘where’, ‘how’, and ‘why’ to engage the researcher’s critical thinking [31]. Examples of these questions may include the following:

I want to find out….

- who has an increased risk of suffering a particular injury?
- what the typical comorbidities are for a particular disease?
- when a particular disorder usually occurs?
- where a particular injury occurs most often?
- how a particular complaint should be treated?
- why it is necessary for the therapist to know and care about a particular clinical problem?

Certain questions present challenges, while others do not. A research problem, which is also considered a conceptual problem, refers to an issue that a researcher aims to resolve by examining pertinent data. Research priorities hold significance in every discipline as they contribute to the advancement of specialized knowledge within specific research areas relevant to a particular field of study [2]. Researchers should demonstrate a willingness to question established beliefs and invest the time necessary to understand the issues surrounding the research problem, rather than jumping directly to finding solutions to specific problems [41]. 

The item ‘Investigable’ assesses whether the research problem can lead to facts, not opinions [48], and is clearly linked to the overall goal of the research topic. Furthermore, it asks whether the study can be conducted based on the identified research problem in terms of clinical trial type and phase, budget, informed consent, sites, resource constraints in terms of personnel and facilities, and timeline [7], as well as the regulatory environment, inadequate patient recruitment, enrollment, and retention [49]. 

The ‘Timely’ item assesses whether the identified research problem is current in the scientific literature and has not already been extensively researched by other authors, when it should be investigated (start of study), how long the research process will realistically take (timeframe of study), including all preparations for data collection, data collection itself, data processing and analysis, and when the results of the research project should be published. This is closely related to the item ‘Investigable’. Depending on the study design, e.g., clinical trial, observational study or systematic review, established checklists, such as the Standard Protocol Items: Recommendations for Interventional Trials (SPIRIT) statement [50], the Strengthening the reporting of observational studies in epidemiology (STROBE) statement [51], or the Preferred Reporting Items for Systematic reviews and Meta-Analyses (PRISMA 2020) statement [52] can be used to guide the steps to be taken to manage the research.

## Implementation of SQUARE-IT

The SQUARE-IT approach can be implemented in the clinical setting, where research problems arise from practical uncertainty. In addition, SQUARE-IT can be used in the educational setting where students need to learn the research process and how to identify and narrow down topics for their thesis. In addition, researchers can use it to systematically derive from the current literature why their identified research problem needs to be addressed and resolved [53], and what objectives and focused clinical questions arise from that research problem. An example, of how SQUARE-IT can help, is suggested in Box 5.

**Table:** 

Box 5. Example of how a student might use SQUARE-IT	
A physical therapy student plans to study the use of home exercise for patients with knee osteoarthritis. Her first idea is to examine barriers to home exercise in this population. She needs to determine whether barriers to home exercise are a research problem and justify the research problem by providing evidence of its relevance. She also needs to describe how her study will provide new knowledge about the research problem. Therefore, she must specify the research problem to focus attention on this specific aspect of the study (S). Then she must evaluate whether the research problem is quantifiable, based on clear independent and dependent variables, by showing how they can be measured using valid and reliable instruments or questionnaires (Q). Next, she must assess whether the research problem is relevant and usable by specific audiences (U), precise enough to clearly define the study objectives by anticipating the research question using PICO(T) (A), and restricted in terms of variables of interest and methodological aspects (R). She must ask herself whether the research problem is eligible in terms of existing evidence, priorities for future research, and her own research interests (E), and whether it can lead to facts, not just opinions, in health care, by choosing the appropriate study design in terms of internal validity (I). She must demonstrate that her identified research problem is current and relevant in the research field and to the target population, and has not been researched previously, by carefully reviewing the literature. She also needs to estimate the research process from preparation, recruitment of participants and data collection to data analysis and reporting of results using a time line, e.g. Gantt chart (T). From there, she can proceed to the final definition of her study objectives and the formulation of her focused clinical question, e.g. using PICO(T), and her hypothesis.	

## Limitations of SQUARE-IT

In the EBP literature, there are many formats, checklists, and statements to guide clinicians, therapists, and researchers in reporting their studies or formulating their clinical question. One might ask if there is a need for another approach to guide researchers. Since research is described as a process that is clearly divided into ‘research topic’, ‘research problem’, ‘aim/objective’, and ‘research question’ [53], the answer is yes. However, it is important to note that SQUARE-IT does not intend to replace or overshadow any of the established guides, checklists, statements, or formats, as each has its place in contributing to successful, transparent, and relevant research efforts. Many of the relevant checklists and Risk of Bias tools have been recently summarized [54]. Examples for checklists, guides and formats are the Consolidated Standards of Reporting Trials (CONSORT) statement [55], the STROBE statement [51], the PREPARE trial guide [10], the REPORT trial guide [56], the PICO-format [8], the FINER approach [12], the SMART-format [57], and the SPIRIT statement [50]. For example, SPIRIT is a checklist for comprehensive reporting of a trial protocol. However, it only addresses the item to include a description of the background and rationale for the planned trial, but it lacks detailed guidance on the key issues in formulating the background and rationale, including identifying the research problem that leads to the focused clinical question. SQUARE-IT can fill this gap, but it cannot distinguish between different types of research problems, i.e., practical and research-based research problems [53]. 

## Further considerations for the SQUARE-IT approach

In addition to elucidating the definition of each SQUARE-IT item, it is crucial to identify not only delineate research questions but also recognize the gaps in existing evidence. This comprehensive understanding is vital to effectively guide researchers, clinicians, policymakers, and funders regarding the specific areas that require investigation and the requisite study designs for addressing those inquiries [22]. Moreover, it is imperative for all stakeholders to have a clear understanding of the specific areas in which the individual research is conducted to improve the results of research evidence in order to best target funding [24]. There are methods for identifying research problems, such as workshops, literature reviews, suggestions submitted through a website, and face-to-face engagement activities, but they are considered time-consuming or labor-intensive and may lack informed expert opinion [58]. Therefore, much stronger evidence is required to determine the effectiveness of methods used to identify research problems, such as Delphi-like surveys involving experts in the field of research and higher education. The SQUARE-IT approach may guide or even replace such Delphi-like surveys, which has to be further studied. Researchers must remain cognizant that studies involving human subjects or animals have the potential to inflict harm, be it physical, psychological, emotional, invasive procedures, treatments, or the risk of embarrassment [31]. Therefore, when identifying a research problem, the researcher should always be concerned that people or animals who will be involved in the research will not be harmed.

## Conclusion

The SQUARE-IT approach constitutes an innovative instrument, intended to aid aspiring, novice scientists and clinicians in the fields of sports medicine and physical therapy in their early scientific careers to formulate clinically relevant research questions, based on meaningful, contemporary research problems. We strongly advocate for the adoption and utilization of this approach, as delineated in this article, while also emphasizing the importance of exploring additional pertinent factors in subsequent publications. Such endeavors will engender a comprehensive discourse and enhance the methodologies of evidence-based practice within the domains of medical and therapeutic specialties. In addition, we propose the possible extension of the SQUARE-IT approach to different disciplines, thus embracing a multidisciplinary framework, and the validation of the approach by recognized consensus methods, such as the Delphi method, and its application to a group of e.g. novice researchers with subsequent evaluation.

## Data Availability

No datasets were generated or analyzed during the current study.
